# The Effect of Message Framing on COVID-19 Vaccination Intentions among the Younger Age Population Groups: Results from an Experimental Study in the Italian Context

**DOI:** 10.3390/vaccines10040559

**Published:** 2022-04-04

**Authors:** Sara Betta, Greta Castellini, Marta Acampora, Serena Barello

**Affiliations:** 1 EngageMinds HUB—Consumer, Food & Health Engagement Research Center, Università Cattolica del Sacro Cuore, 20123 Milan, Italy; sara.betta01@icatt.it (S.B.); marta.acampora01@icatt.it (M.A.); serena.barello@unicatt.it (S.B.); 2 Faculty of Agriculture, Food and Environmental Sciences, Università Cattolica del Sacro Cuore, Via Milano 24, 26100 Cremona, Italy; 3 Department of Psychology, Università Cattolica del Sacro Cuore, L.go Gemelli 1, 20123 Milan, Italy

**Keywords:** framing effect, COVID-19 vaccine, health communication, vaccine hesitancy, vaccine acceptance

## Abstract

The coronavirus pandemic has been recognized as a major threat to public health. Widespread acceptance of COVID-19 vaccines is crucial for achieving adequate immunization coverage to end the global pandemic. However, to date, there are still hesitant people, especially among the younger population groups. For this reason, it is essential to identify the psychological variables that may affect vaccination intention among these “at risk groups” and to select possible successful communication frames in order to increase vaccination rates. An online cross-sectional survey was carried out on 208 Italian citizens younger than 50 years old, to explore message framing effects on their intention to receive the vaccination and to understand the psychological factors influencing it. Results showed that depending on the goal (stimulate vaccination intention, vaccination trust, or vaccine attitude), not all the communication stimuli are equally effective on this target population. Furthermore, the study showed that sociodemographic variables do not help to explain the vaccination intention of the younger population groups, while the psychological variables have been found to have a stronger impact on such attitude. Trust and attitudes towards vaccines, concern about the pandemic and concern about infecting others have been found to be the most effective predictive variables of people vaccination intention. The study results underline the importance of understanding the psychological roots behind vaccine hesitancy to shape sensitization actions and vaccination campaigns targeting this population group.

## 1. Introduction

Immunization programs have significantly reduced infectious diseases, preventing infections or reducing the severity of symptoms and have increased public health standards by reducing morbidity and mortality rates [1]. However, vaccination campaigns can be effective in the long run—if accepted by large segments of the population. The response to a vaccine can be understood as a continuum ranging from absolute rejection to actively demanding immediate uptake [2]. Vaccine hesitancy, defined as the unwillingness to be vaccinated when a vaccine is available [3], has been identified by the World Health Organization as one of the top ten threats to global health in 2019 [4]. Large variability in COVID-19 vaccine acceptance rates has been reported in different countries and areas of the world [5]. A large number of studies reported COVID-19 acceptance rates below 60%, which would represent a serious problem for virus control efforts [6]. In the COVID-19 pandemic, as with future epidemics of vaccine-preventable diseases, it will be important to address this resistance by promoting vaccine uptake through effective communication strategies [7]. Previous studies [6,8,9,10] have investigated whether different individual characteristics, such as age, sex, and living conditions, influence willingness to get vaccinated. According to a large multinational study analyzing COVID-19 vaccine acceptance across 15 survey samples covering 10 low- and middle-income countries (LMICs) in Asia, Africa, South America, Russia (an upper-middle-income country) and the United States, including a total of 44,260 individuals. Lower-income individuals and women are less prone to getting vaccinated against COVID-19 [11,12]. Regarding age groups, many studies conducted during the COVID-19 pandemic showed that younger age population groups (aged less than 50 years old) seems to be less motivated to get vaccinated because they perceive their health-related risk to be lower than older group populations [12,13,14]. Many countries have experienced higher vaccine hesitancy among younger generations, slowing the progress of vaccination campaigns [15,16,17,18,19]. Moreover, younger generations have become primary drivers of the spread of COVID-19 [20], and thus they are a relevant target group for public health communication campaign aimed at fostering vaccination behaviours.

A well-established messaging strategy to promote vaccination behaviours is health message framing [21]. Behind these theory, there is the assumption that the way people process health-related information is not completely rational [22]. This means that people decision-making preferences are also affected by how information is presented and through which source [23,24]. Therefore, understanding the process of interaction between information and individuals and designing effective messages to influence individuals’ decision-making processes can produce a positive impact when communicating during a public health crisis like the COVID-19 pandemic. According to this theory, to increase people’s intention to get vaccinated and reduce their level of vaccine hesitancy, it is possible to use different types of message Framing—Basing on specific motivational roots behind individuals’ vaccination intentions. Based on previous studies [25,26], the most relevant motivations that may lead people intention to get vaccinated are related to three main issues: personal health risks (i.e., the possibility of getting seriously sick), economic costs (i.e., the financial burdens associated with the economy “shutting down” in order to contain the virus’ spread), and/or the collective public health consequences (i.e., the possibility of infecting others; including vulnerable populations) [7].

Furthermore, these issues, when communicated in public health campaigns, may change in their perceived relevance depending on the communication source (e.g., whether messages originate from medical experts vs. lay influencers) [7].

According to these premises, this study was aimed to explore the influence of message framing on COVID-19 vaccination intention and provide a scientific basis for the communication plan to improve vaccination rate among the younger groups of the Italian population.

In particular, this paper reports the results of a cross-sectional study whose specific aims were: (1) to explore information framing effect on the younger publics’ intention to receive the COVID-19 vaccination and (2) to understand the key psychosocial factors influencing the intention to get the COVID-19 vaccinations in this population. As misinformation and uncertainty have infiltrated the public space, this study aims to provide useful knowledge for public agencies about how to communicate effectively with the younger public to wade through fake news and maximize public health communication campaign effectiveness.

## 2. Methods

### 2.1. Participants

To reach the study aims, an online experiment was conducted. Four hundred and five surveys were collected, of which 208 were analyzed as they were fully completed. In particular, the sample was composed by 208 Italians, aged under 50 years old, recruited through a convenience sampling. The questions were presented in an online survey, delivered using Qualtrics software (Seattle, WA, USA). The weblink to administer the survey was distributed through social networks such as Facebook, LinkedIn and WhatsApp basing on the researchers’ personal networks’ contacts. Inclusion criteria were: (1) being not vaccinated with COVID-19 vaccine (2) being aged between 18 and 50 years old and (3) informed consent and voluntary participation to the study. Exclusion criteria were: (1) refusing to participate in this study and (2) not speaking Italian language. The study survey was conducted using a CAWI (Computer Assisted Web Interviewing) methodology between May and June 2021. This study has been performed in accordance with the Declaration of Helsinki and has been approved by an independent ethical commission of the Department of Psychology-Università Cattolica del Sacro Cuore in Milan (CERPS) (Approval Code: IRB#02-20).

### 2.2. Design and Measures

The study used a within-subject factorial design experiment with 6 conditions including two crossing factors: message frame (3 conditions: personal health risks, collective public health consequences of not vaccinating, and economic costs) and message source (2 conditions: virologist as an expert source or influencer as a lay source). Regarding the expert source of information, we decided to select the virologist as, in Italy, these professionals were mostly in charge of communicating decisions regarding the vaccination campaign both leveraging on health-related and economic-related motivations. Regarding the lay source, we decided to select Italian famous influencer—i.e., Chiara Ferragni and Fedez —As during the pandemic they were involved in public campaigns to sustain the vaccination behaviours. After reading the informed consent and having agreed to participate, participants provided socio-demographic information (i.e., age, gender, profession, and education). Then, all participants were shown six written communicative stimuli by crossing the 2 experimental factors, i.e., the message frame (personal health risks, collective public health consequences of not vaccinating, and economic costs) and the message source (virologist as an expert source or influencer as a lay source) (Table 1 reports the communication frames used in the study survey translated in English for publication purposes).

In detail, the research design is composed of 6 conditions for each of which a different communication frame regarding vaccines was shown to each participant (condition 1: virologist/personal risk; condition 2: influencer/personal risk; condition 3: virologist/risk to the collective health; condition 4: influencer/risk to the collective health; condition 5: virologist/economic risk; condition 6 influencer/economic risk). These communication frames were presented randomly, to control for sequence and order effects, to all participants. For each condition, participants reported their intention to receive the COVID-19 vaccine, their trust in and their attitudes towards vaccines in general.

Using a within-subject factorial design experiment (where all subjects see all frames) it is possible to compare the responses given by the subjects and understand if the vaccination intention, their level of trust and their general attitudes change (i.e., increase or decrease) when the presented frames change its characteristics in term of contents and source.

In particular, the intention to vaccinate against COVID-19 was measured with an ad hoc item on a response scale from 0% to 100% (where 0 = definitely not, and 100 = definitely yes). Overall trust in vaccines was measured with an ad hoc item assessed on a four-point Likert scale (where 1 = disagree to 4 = completely). Finally, attitude towards vaccines was evaluated using five items that were taken from the 5C scale [27] and adapted to this study and were measured on a seven-step Likert scale (where 1 = strongly disagree and 7 = strongly agree).

In the last part of the survey, the subjects were asked to answer questions useful to profile them from a psychological point of view. Validated scales and item ad hoc were used to measure the followings:

- The level of COVID-19 concern: three items ad hoc were used to assess the level of concern towards (1) COVID-19 emergency (“*How much are you concerned about the COVID-19 emergency?*”); (2) the risk to be infected by COVID-19 (“*How much are you concerned about the risk of being infected?*”); (3) the risk of infecting others (“*How much are you worried about the risk of infecting other people?*”). These items were measured on a 10-point scale (0 = not concerned at all; 10 = very concerned).
- The level of Health Engagement: the first item of the Public Health Engagement Scale for Emergency Settings (PHEs-E) [28] was adopted to assess the readiness of individuals to adhere to the public health prescriptions to control the virus spread. The item was measured on a 7-point scale (1 = *I′m in a panic*; 7 = *I feel in control*).
- The level of conspiracy mentality [29] was measured through an adaptation of the Generic Conspiracist Beliefs Scale [27]. Five items were adapted to this study and measured on a scale from 1 (absolutely not) to 100 (absolutely yes). An example of item is “*I think that many important things happen in the world that people are not informed about*”. The scale showed a very good reliability (Cronbach Alpha of 0.824).

The survey was conducted using the Qualtrics^®^ (Seattle, WA, USA) software e [30], and it took approximately 15 min to be completed.

### 2.3. Data Analysis

To test the impact of message frame and message source on the intention to vaccinate against COVID-19, Trust in vaccines and Attitudes towards vaccines, a 3 (frame) × 2 (message source) repeated-measures analysis of variance (ANOVA) for each of the three dependent variables was conducted. A Bonferroni post-hoc was used to conduct pair-wise comparisons of means when a significance (*p* < 0.05) was detected, and the main effects and interactions were studied at the significance level *p* = 0.05. If Mauchly’s Test indicated a violation of sphericity, Greenhouse-Geisser correction was used and ε was reported. Moreover, a hierarchical regression to identify the determinants of vaccination intention towards the COVID-19 vaccine was carried out.

By setting the vaccination intention as the dependent variable, the various independent variables were inserted step by step. In the first block, the variables that were assumed to be less explanatory than the intention were included, that is the socio-demographic variables: gender (0 = male; 1 = female), profession and educational level. In particular, the level of education was treated considering the subjects as graduates (0) or non-graduates (1) and the profession variable was reduced to two levels, students (0) and workers (1). Subsequently, in the second block, the questions on COVID-19 concern were added with the related three ad hoc items. In the third block, the variables that were presumed to be the most explanatory of the participants’ vaccination intention were included: trust, attitudes towards vaccines in general, conspiracy and health engagement. In particular, the variables concerning the trust and attitudes towards the vaccine were calculated by averaging the responses given by the subjects for the different communication frames. A *p*-value less than 0.05 was considered statistically significant. All statistical analyses were performed with SPSS Statistics (Version 26, IBM Corporation, New York, NY, USA).

## 3. Results

The sample consisted of 208 people (79.7% females), aged between 19 and 42 years (M = 26.75, SD = 4.62). Table 2 shows in more details the demographic and psychosocial characteristics of the sample.

Considering the COVID-19 related concerns, this study showed that respondents tend to be more concerned about the risk of infecting other people (77.9%) rather than being infected (45.2%). Regarding the variable of health engagement, study participants reported to “try to stay calm” in 62.5% of cases and the18.3% reported “they feel they are in control”. With regards to conspiracy attitudes, respondents, on average, are more likely to believe that “many important things happen to the world that the population is not informed of” (average response = 72.5) and that “politicians often do not tell us the real reasons behind their decisions “(average response = 72.1) compared to believing that” events that superficially appear to be unrelated are often the result of secret activities “(average = 37.6).

### 3.1. Impact of the Source of Message (Virologist or Influencer) and Type of Communication Frame (Personal Health Risks, Collective Public Health, and Economic Costs) on the Intention to Receive the COVID-19 Vaccine

Since Mauchly’s Test indicated a violation of sphericity about frame, χ^2^(2) = 38.48, *p* < 0.001, Greenhouse-Geisser correction was used (ɛ = 0.85). We did not find a main effect of the source (virologist or influencer) on the intention to receive the COVID-19 vaccine, F(1, 207) = 3.265, *p* > 0.05, ηp2 = 0.016, and also the different types of frame (personal health risks, collective public health, and economic costs) do not have a main effect on the intention to receive the COVID-19 vaccine F(1.71, 353.72) = 2.200, *p* > 0.05, ηp2 = 0.011. On the contrary, the study results showed a significant interaction (*p* < 0.05) between frame and source F(2, 414) = 3.204, *p* = 0.042, ηp2 = 0.015). Table 3 showed that the intention to receive the COVID-19 vaccine increases if the message frame deals with the personal health risk and the source of the message is a virologist and not an influencer (M = 85.52, SD = 26.03; M = 84.10, SD = 26.65; respectively with *p* = 0.039); the same results could be observed for messages that are focused on the economic costs related to being not vaccinated (M = 85.21, SD = 27.13; M = 83.94, SD = 27.23; respectively with *p* = 0.044). Conversely, the intention to get the vaccine does not change significantly if it is used a virologist or an influencer to give the message focused on collective public health (M = 85.38, SD = 26.41; M = 85.73, SD = 25.78; respectively with *p* = 0.531).

### 3.2. Impact of the Source of Message (Virologist or Influencer) and Type of Communcation Frame (Personal Health Risks, Collective Public Health, and Economic Costs) on the Trust in Vaccines

Since Mauchly’s Test indicated a violation of sphericity about frame, χ^2^(2) = 17.39, *p* < 0.001, Greenhouse-Geisser correction was used (ɛ = 0.93). We did not find a main effect of the source (virologist or influencer) on the trust in vaccines, F(1, 207) = 0.332, *p* = 0.565, ηp2 = 0.002, and also the different types of frames (personal health risks, collective public health, and economic costs) do not have a main effect on the participants’ trust in vaccines F(1.85, 382.99) = 1.283, *p* = 0.277, ηp2 = 0.006. We did not find a significant interaction between frame and source F(2, 414) = 1.064, *p* = 0.346, ηp2 = 0.005) (Table 4).

### 3.3. Impact of the Source of Message (Virologist or Influencer) and Type of Communication Frame (Personal Health Risks, Collective Public Health, and Economic Costs) on the Attitudes towards Vaccines

Since Mauchly’s Test indicated a violation of sphericity about source*frame, χ^2^(2) = 9.576, *p* < 0.001, Greenhouse-Geisser correction was used (ɛ = 0.96). We did not find a main effect of the source (virologist or influencer) on the Attitudes towards vaccines, F(1, 207) = 0.143, *p* = 0.706, ηp2 = 0.001, and also the different types of frame (personal health risks, collective public health, and economic costs) do not have a main effect on the Attitudes towards vaccines F(2, 414) = 0.066, *p* = 0.936, ηp2 = 0.000. We did not find a significant interaction between frame and source F(1.91, 396.01) = 1.785, *p* = 0.171, ηp2 = 0.009) (Table 5).

### 3.4. Predictors of Vaccination Intention in Young People

A Hierarchical Regression was carried out to identify the determinants of vaccination intention against the COVID-19 vaccine in the study respondents (Table 6). Model 1 reports nonsignificant results. Model 2 included the COVID-related concern variables in its three levels (concern about the emergency, concern about being infected and concern about infecting others) as an additional factor. Adding the variables of concern to the models increased the explained variance by 2–15.9%, making a significant contribution to the prediction of the study participants’ vaccination intention (*p* < 0.001). Adding the psychological variables Health Engagement, Conspiracy, General Attitude toward vaccines (higher scores on this scale indicate high levels of positive vaccine evaluation) and General Trust in vaccines (higher scores on this scale indicate high levels of trust in vaccines) improved the Model 3 and increased the explained variance by 15.9–33.1%, making a significant contribution to the prediction of the vaccination intention (*p* < 0.001). Considering the last model (model 3) including all variables, it is possible to observe that the overall regression was statistically significant (R^2^ = 0.509, F(10, 197) = 20.421, *p* < 0.001). It was found that the concern for the emergency (β = 0.226, *p* < 0.001), the concern of infecting others (β = 0.126, *p* < 0.05), the general attitude toward vaccines (β = 0.225, *p* < 0.01) and the general trust in vaccines (β = 0.447, *p* < 0.001) significantly predicted the participants’ intention to vaccinate. In particular, the General trust in vaccine is the variable that most affects vaccination intention. There are no problems of collinearity between the variables as all the VIF values ranged from 1.039 to 1.989 and the Tolerance between 0.503 to 0.963 [31].

## 4. Discussion

This study was aimed to explore the influence of message framing on populations younger than 50 years old on their COVID-19 vaccination intention and provide a scientific basis for public health communication strategies to improve vaccination rates. To achieve this aim, an online survey was developed and administered to a sample of Italian citizens aged between 19 and 42. All participants were shown six communicative written stimuli by crossing two variables: the communication frame and the source of the message. Then, after seeing each communication frame, they were asked to report their intentions to receive the COVID-19 vaccine and their overall trust and attitudes towards the vaccine. Finally, the subjects were asked to answer questions to describe them from a psychological point of view.

Regarding the first research aim, which was to explore the effect of information framing on participants’ intention to receive COVID-19 vaccination, overall trust and attitude toward vaccines, the study showed that the different frames do not have a significant effect on vaccine trust and general vaccine attitude, while influencing the intention to vaccinate against COVID-19.

In particular, if the purpose is to increase younger population groups’ intention to get vaccinated against COVID-19, it is important to underline that depending on the selected frame, a specific message source will be better indicated. Specifically, if a message emphasizes the personal risks due to lack of vaccination, this study recommends using the virologist as a source. The same is for messages that is focused on the economic risk associated with not being vaccinated. On the other side, the impact of focusing the message of collective health risks due to lack of vaccination does not differ according to the source of the message: research results showed that there are no significant differences between the source in this case. These results confirm some previous studies that demonstrated the effect of the “principle of authority” arguing that as human beings, we tend to have more trust in experts or figures that we perceive to be more authoritative in certain contexts or subjects. According to these studies, people are willing to believe in people who we recognize have competence and credibility towards a topic, thus highlighting that medical consensus on vaccine safety is effective in decreasing people′s hesitancy about vaccination behaviours [32,33].

Considering the second main aim of this research related to understanding the key psychosocial factors influencing the intention of COVID-19 vaccinations in our study population, results showed that the socio-demographic variables (gender, educational level and profession) do not explain the vaccination intention of our population group. On the contrary, concerns regarding the pandemic, being infected and infecting others are paramount to understand this phenomenon. However, the psychological variables (trust and attitudes toward vaccines, level of conspiracy and health engagement) have the greatest impact on the participants’ vaccination intention and best explain the variability of this phenomenon. In particular, the concern about the emergency caused by the pandemic, the concern about the risk of infecting other people and the overall trust and attitude towards vaccines positively affect the study participants’ intention to vaccinate against COVID-19. These results are in line with previous studies [34,35] that showed that the younger population perception of personal health risk (such as the worry of being infected) is not a determining factor for their intention to vaccinate against COVID-19 [31]. The same studies highlight that for the younger population groups are more motivated by their concern for the emergency in general and above all by the fear of infecting other people. Again, our results are in line with other studies that showed that differently from the elderly population that has been showed to accept vaccination due to their perceived risk of developing serious illness and concerns about being infected, younger people accept vaccines for reasons related to psychological factors, such as trust in vaccines and attitudes towards them [32,36,37,38,39,40]. In summary, the results of this study contribute to provide theoretical and practical suggestions to inform communication campaign that consider the psychosocial roots behind vaccination behaviour. These results show that the predictors of vaccination intention are subjective aspects strongly connected with the psychological characteristics of people. The pandemic, in addition to its evident health and economic implications, has had very important consequences on the psychological and social level. For this reason, as other studies suggested [41,42,43], to identify the psychological roots of vaccine hesitancy and / or intention in this specific context is an essential prerequisite for achieving and maintaining high vaccination rates, as well as for outlining educational paths and campaigns to increase their acceptance. Therefore, it is possible to state that only a personalized communication based on a deep understanding of the psychological reasons behind the intention to get vaccinated, can help experts to enhance and encourage vaccination in the younger population groups.

### Future Research and Limitation

This research results should be interpreted with caution because of some study limitations. First, the sample is not representative of the study population. Therefore, it is not possible to extend these results to the younger groups of the Italian population. Furthermore, the study sample could be affected by self-selection bias due to the recruitment process and to the fact that people that accepted to participate to the study could have different characteristics from the ones who declined their participation. Unfortunately, we did not collect information about those who refused to participate and thus we cannot compare characteristics of respondents and non-respondents. Moreover, the communication frames took into consideration only two cross-over variables, namely the type of source, and the content of the message without considering other possible interesting variables such as the communication channel of the message (e.g., radio, TV, social networks). We suggest considering these aspects in future study to address this research gaps. Moreover, it would be interesting to investigate the specific communication frame conditions that foster older population groups to get vaccinated against COVID-19 and compare them with those that emerged as effective in the younger population. This would contribute to develop customized communication campaigns according to the target populations. Finally, this study was carried out only in Italy. This should be considered as a possible influencing variable that may have affected results. Further cross-cultural studies should be conducted to explore how cultural aspects could affect this population segment’s vaccination behaviours. This study was conducted in the late spring of 2021 regarding attitudes towards the COVID-19 vaccine in the Italian younger population (when the vaccine in Italy had just been made available to this age group), but the implications are also transferable to promoting the acceptability of future vaccines.

## Figures and Tables

**Table 1:** Message frames administered in the study survey.

Type of Frame	Virologist	Influencer
**Personal Risk**	“Are you thinking of not getting the COVID-19 vaccine? You are risking your health”! So declares Professor Benati, a virologist at the Ferravalle Hospital. “According to our research -the professor adds-, people who do not get vaccinated against COVID-19 have a high risk of contracting several long-term health complications. With a disease like COVID-19, the risk should not only be assessed in terms of the number of deaths; in fact, we talk about a multisystem disease, which can cause damage to various organs of the body. moreover, there is no evidence on the long-term scientific effects of COVID. Our research is leading to show that a 30-year-old is more likely to have long-term consequences after COVID (such as chronic fatigue, shortness of breath, gastrointestinal problems, etc.) than the risk of death for a 60-year-old. So, for this reason, it is essential that all young people for whom there are no medical indications, get the vaccine as soon as possible!”	“Are you thinking of not getting the COVID-19 vaccine? Ask someone who has contracted the virus! “ So declare, Fedez and Chiara Ferragni. “This afternoon- add the Ferragnez- Marco, a 28-year-old boy, wrote to us and we want to share his experience with all of you. Oh yes, his quarantine began in October, among illness, COVID swab and the hope of being told “it′s all over”. Marco told us that at the beginning he was sure that everything would last a few days and instead when the classic symptoms disappeared, the indelible signs of COVID showed up: headache, joint pain, exhaustion and cough. Endless days of confinement in which you feel like you′re on a swing, between moments of apparent well-being and states of total discomfort. We hope, as the Ferragnez say, that this experience will be a warning to everyone. This is not an ordinary flu but a disease that leaves its marks even in the long term. So, for this very reason, it is essential that all young people for whom there are no medical indications, get the vaccine as soon as it is their turn!
**Risk To The Collective Health**	“Are you thinking of not getting the COVID-19 vaccine? Prepare for more deaths and hospitalizations ”! So declares Professor Miravalle, virologist at the Ripali Hospital. “According to our research- adds the Professor- at least 3 out of 4 Italians must receive the COVID-19 vaccine to reduce the spread of the new coronavirus and bring mortality and hospitalization rates down to pre-pandemic levels. Out of 4 Italians choose not to receive the vaccine, mortality and hospitalizations will continue to increase and this means that we will not be able to achieve herd immunity as many Italians will continue to contract and spread COVID-19. If we want to end this pandemic, we need young people to get vaccinated too. So, for this very reason, it is essential that all young people, for whom there are no medical indications, get the vaccine as soon as it is their turn!”	“Are you thinking of not getting the COVID-19 vaccine? Tell the people who depend on your choice not to get sick “! So declare Chiara Ferragni and Fedez. “In these days, -add the Ferragnez-Sofia, a law student currently undergoing chemotherapy treatments to fight leukemia, wrote to us. Since she cannot get the COVID-19 vaccine and therefore has a higher risk of contracting COVID-19 in severe forms, which is why her health depends largely on the health of others! By vaccinating, we will be able to stop the spread of COVID-19. This reduces the chances that people like Sofia, who cannot develop antibodies to the virus, will get sick. So, it is essential that all young people, who are not against medical indications, get the vaccine as soon as it is their turn!
**Economic Risk**	“Are you thinking of not getting the COVID-19 vaccine? Get ready for a slower economic recovery ”! So declares Professor Rissori, virologist at the Piemmolo Hospital. “According to our research- adds the Professor- to ensure a rapid economic recovery at least 3 out of 4 Italians must receive the COVID-19 vaccine. If more than 1 in 4 Italians choose not to receive the vaccine, Italy will be forced to continue the gradual closure of activities to stop the spread of the virus. This could cause millions of Italians to lose their jobs. If we want to put an end to the economic difficulties that this pandemic has unleashed, we need Italians to get vaccinated. So, for this very reason, it is essential that all young people for whom there are no medical indications, get the vaccine as soon as it is their turn!”	“Are you thinking of not getting the COVID-19 vaccine? Tell someone who lost their job ”! So declare Chiara Ferragni and Fedez. This afternoon, the Ferragnezes add, “Luca, 27, a graduate in Economics and Management, wrote to us, who lost his job due to the coronavirus last March. Although his company was able to allow some employees to work from home, he was one of the unfortunates few who lost their jobs due to massive budget cuts as the newcomer. Luca barely has enough money set aside to pay the rent and this situation forced him to return to live at home with his parents asking them for support. Although he is actively looking for a new job, there are simply not many opportunities. If a sufficient number of people decide to get vaccinated, we can stop the spread of COVID-19 and start the economy again and avoid consequences like the one described by Luca. So, for this very reason, it is essential that all young people for whom there are no medical indications, get the vaccine as soon as it is their turn!”

**Table 2:** Demographic and psychosocial characteristics of the sample (n = 208).

	n	%
**1.** **Gender**		
Males	61	29.3
Femalesz	147	70.7
**2.** **Age**		
19–30	184	88.3
31–42	24	11.7
**3.** **Geographic area**		
North	147	70.6
Center	24	11.5
South	37	17.8
**4.** **Profession**		
Student	108	51.9
Worker	100	48.1
**5.** **Education**		
Before Graduation	58	27.9
After Graduation	150	72.1
**6.** **Marital Status**		
Single	174	83.7
Married/Cohabitant	34	16.3
**7.** **Concern for the epidemic** (M = 7.3; SD = 2.1)		
Low (1–3)	15	7.2
Medium (4–7)	69	33.2
High (8–10)	124	59.6
**8.** **Concern of being infected** (M = 5.9;SD = 2.9)		
Low (1–3)	36	17.3
Medium (4–7)	78	37.5
High (8–10)	94	45.2
**9.** **Concern of infecting others** (M = 8.5;SD = 1.9)		
Low (1–3)	7	3.4
Medium (4–7)	39	18.7
High (8–10)	162	77.9
**10.** **Level of Health Engagement**		
I am in panic	3	1.4
2	1	0.5
I feel the urge to do something	23	11.1
4	6	2.9
I try to stay calm	130	62.5
6	7	3.4
I feel in control	38	18.3
**11.** **Conspiracy**		
I think that many important things happen in the world that people are not informed about	(M = 72.5; SD = 23.4)	
politicians often do not tell us the real reasons behind their decisions	(M = 72.1; SD = 25.8)	
events that superficially appear to be unrelated are often the result of secret activities	(M = 37.6; SD = 28.9)	
I think there are secret organizations that exert an important influence on political decisions	(M = 41.6; SD = 31.5)	
I think government agencies closely monitor citizens	(M = 48.2; SD = 28.2)	

Note: Geographic area: North (Liguria, Lombardia, Piemonte, Valle d′Aosta, Emilia-Romagna, Friuli-Venezia Giulia, Trentino-Alto Adige and Veneto); Center (Lazio, Marche, Toscana and Umbria); South (Abruzzo, Basilicata, Calabria, Campania, Molise, Puglia, Sardegna and Sicilia); Education level: Before graduation (no educational qualification, primary school, secondary school, professional qualification, high school), After Graduation (bachelor degree, master degree, PhD degree); M = mean, SD = Standard Deviation.

**Table 3:** Overall intention to receive the COVID-19 vaccine.

	Personal Health Risks	Collective Public Health	Economic Costs	Average Intention to Receive the COVID-19 Vaccine
Virologist	85.52 ^a^ A	85.38 ^a^ A	85.21 ^a^ A	**85.37 A**
Influencer	84.10 ^a,b^ B	85.73 ^a^ A	83.94 ^b^ B	**84.59 A**
Average intention to receive the COVID-19 vaccine	**84.81 ^a,b^**	**85.55 ^a^**	**84.58 ^b^**	**-**

Note: n = 208; different lowercase and uppercase letters identify significant differences between means in column and in row, respectively.

**Table 4:** Overall trust in vaccines.

	Personal Health Risks	Collective Public Health	Economic Costs	Average Trust in Vaccines
Virologist	3.27	3.27	3.25	**3.27**
Influencer	3.28	3.24	3.25	**3.26**
Average trust in vaccines	**3.28**	**3.26**	**3.26**	**-**

Note: n = 208.

**Table 5:** Overall Attitudes towards vaccines.

	Personal Health Risks	Collective Public Health	Economic Costs	Average Attitudes towards Vaccines
Virologist	5.95	5.99	5.99	**5.98**
Influencer	6.01	5.96	5.98	**5.98**
Average Attitudes towards vaccines	**5.98**	**5.98**	**5.99**	**-**

Note: n = 208.

**Table 6:** Hierarchical regression analysis predictors of intention to receive the COVID-19 vaccine.

Variable	Model 1			Model 2			Model 3		
	B(se)	β	*p*-Value	B(se)	β	*p*-Value	B(se)	β	*p*-Value
**Socio-demographic**									
**Gender**	4.860 (4.004)	0.086	0.226	−2.262 (3.981)	−0.040	0.571	−1.116 (3.122)	−0.011	0.837
**Educational level** **(low; high)**	−0.590 (4.020)	−0.010	0.883	−2.594 (3.723)	−0.045	0.487	−1.960 (2.925)	−0.034	0.510
**Profession** **(student; worker)**	−4.817 (3.666)	−0.093	0.190	−3.541 (3.433)	−0.069	0.303	0.763 (2.714)	0.016	0.766
**Concern**									
**for the epidemic**				4.531 (0.945)	0.371	**0.000**	2.742 (0.761)	0.225	**0.000**
**of being infected**				−0.712 (0.749)	−0.080	0.342	−0.956 (0.608)	−0.109	0.113
**of infecting others**				2.343 (1.011)	0.173	**0.021**	1.711 (0.797)	0.126	**0.034**
**Psychological variables**									
**Trust in vaccine**							17.789 (2.803)	0.447	**0.000**
**Attitude towards vaccine**							5.216 (1.629)	0.225	**0.002**
**Conspiracy**							0.064 (0.065)	0.053	0.326
**Health Engagement**							−0.297 (1.192)	−0.014	0.803
**Constant**	64.846 (19.780)		0.001	50.246 (19.110)		0.009	−29.118 (19.735)		0.142
**Model value**	F(3, 204) = 1.317, *p* = 0.256, R^2^ = 0.02, R^2^_Adjusted_ = 0.005			F(6, 201) = 7.244, *p* < 0.001, R^2^ = 0.18, R^2^_Adjusted_ = 0.15			F(10, 197) = 20.421, *p* < 0.001, R^2^ = 0.51, R^2^_Adjusted_ = 0.48		
**Variation (∆R^2^; *p*-value)**	0.02; 0.270			0.159; *p* < 0.001			0.331; *p* < 0.001		

## Data Availability

The datasets used and/or analyzed during the current study are available from the corresponding author on reasonable request.
